# A flow equilibrium model controlling cytoplasmic transition metal cation pools and preventing mis-metalation as exemplified for zinc homeostasis

**DOI:** 10.1128/jb.00228-25

**Published:** 2025-10-09

**Authors:** Dietrich H. Nies

**Affiliations:** 1 Institute for Biology/Microbiology, Martin-Luther-University Halle-Wittenberg 9176, Halle (Saale), Germany; University of Florida, Gainesville, Florida, USA

**Keywords:** *Cupriavidus metallidurans*, metal homeostasis, cobalt, zinc

## Abstract

The metal cations of the first transition period fill up their 3d orbitals from 3d^5^ for Mn(II) to 3d^10^ for Zn(II). Enzymes use these cations as cofactors and exploit their individual chemical features for important catalytic reactions. A prerequisite for this process is metalation of the respective enzyme with the correct cation to form metal complexes, despite the presence of other competing transition metal cations. The first step to avoid mis-metalation requires maintenance of cytoplasmic cation homeostasis, which adjusts not only the concentration of an individual cation but also that of the overall metal-ion pools. This is achieved via a flow equilibrium of metal cation uptake by importers with broad substrate specificity combined with export of unwanted cations by efflux systems. A third group of cation importers with high substrate affinity contributes under metal starvation conditions. Experimental evidence for the existence of such a flow equilibrium comes from studies using the metal-resistant beta-proteobacterium *Cupriavidus metallidurans*. Central to the calibration of the pool of an individual metal cation are the regulators that control expression of the genes for the import and export pumps. A theoretical model that deduces how metal-cation discrimination may be performed by the respective regulator and the pathway from uptake of an external cation to correct metalation provides new insight into these processes.

## HARD, SOFT, AND BORDERLINE TRANSITION METAL CATIONS: WHY SOME ARE USED AND OTHERS ARE NOT

Living cells can only use resources that are available. In terms of transition metals, these are represented by the first row of the transition elements, from Mn to Zn, and the anions of V, Mo, W, Ag, Cd, Au, and Hg (1), but not all of these metals have a beneficial effect and are used. Transition metal cations are Lewis acids, able to interact with Lewis bases, anions, or compounds with a free electron pair. Divalent metal cations in aqueous solutions stay in the vicinity of a Lewis base as contact ion pairs or solvent-shared ion pairs, according to the Debye-Hückel law (2, 3) . Alternatively, they form metal complexes with the Lewis bases as ligands, in most cases with O, N, or S atoms in the first shell. Ligands could be part of an amino acid residue or a cofactor, such as a heme compound. Fe(II), Mn(II), and Cu(II) complexes are redox-active under physiological conditions. Ni(II) and Co(II) ions may change their oxidation state only within a complex, while Zn(II) complexes are redox inactive (Table 1).

**TABLE 1:** Features of transition metal cations^*a*^

Metal	Electrons	Cpl.^*b*^	Ionic radius (Å)	Bond energy (kJ/mol)				Ratio	Charact.
	(4d) 3d		Monovalent, divalent	O	Ionic O (%)	S	Ionic S (%)	S/O	
**Mn(II**)	5	oc	0.80	−36.8	63	−43.9	22	*1.19*	*Hard*
**Fe(II**)	6	oc	0.76	−42.8	51	−53.4	12	1.25	Interm.
Co(II)	7	*oc*	0.74	−45.2	51	−61.8	12	1.37	Interm.
Ni(II)	8	dsq	0.72	−45.8	51	−59.5	12	1.30	Interm.
**Cu(II**)	9	tet	(**0.96**) 0.69	−54.5	47	−104.7	9	1.92	Int/soft
*Ag(I*)	(10)	tet	(**1.26**)	−22.3	47	−145.8	9	**6.53**	**Soft**
*Zn(II*)	10	tet	0.74	−47.4	59	−62.6	19	1.32	Interm.
*Cd(II*)	(10)	tet	**0.97**	−39.7	55	−81.2	15	**2.04**	**Soft**

^*a*^ Features important for the discrimination of the divalent metal cations of the first row of the transition elements from Mn(II) to Zn(II) are shown, additionally Ag(I) as a proxy for Cu(I) plus Cd(II) of the second period. Cations that can change their redox state are in bold letters, whereas those which are redox inert under physiological conditions are in italics. Co(II) and Ni(II) can do this only within metal complexes. The number of d-electrons in the 3d- or 4d-shell, respectively, increases from 5 (a half-filled 3d orbital) to 10 (completely filled orbitals), which subsequently determines possible metal complexes.

^*b*^ These conplexes (Cpl.) are octahedral (oc), distorted octahedral or square planar (dsq) or tetrahedral (tet). For cobalt, an octahedral geometry is stable for the oxidation state Co(III) (italics, gray field). The ionic radii from Mn(II) to Zn(II) are similar, but Cu(I), Ag(I) and Cd(II) are much larger. Since ∆G = −RT ln(K), the bond energy with oxygen and sulfur atoms in hydroxide and sulfide complexes could be calculated from the stability constants of these compounds (4), respectively, and is shown in kJ/mol, plus the percentage of the ionic character of the bond as calculated from the electronegativity according to Linus Pauling. The ratio of the bond energies S/O in combination with the ionic radius indicates the soft (bold letters), hard (italics) or intermediate (interm.) character (Charact.) of the respective ions. Cu(II) (gray field) usually appears as Cu(I) within the reducing environment of the bacterial cytoplasm and is here soft while Cu(II) is intermediate (int/soft). More details are published (1).

The benefit of using transition metal complexes is countered by their toxicity, which originates from mis-metalation of metal-binding sites, uncontrolled redox processes with reactive oxygen species, or binding to thiol residues with subsequent disturbance of either the cellular redox homeostasis or protein conformations (5–19). The metals of the second and third transition periods are cations, whose large-volume electron orbitals and comparably high electronegativity (20–22) result in them binding strongly to sulfur atoms of thiol groups. This leads to a high toxicity of these “soft” cations, which severely limits their usefulness. Exceptions are the oxyanions of Mo and W that are firmly sequestered by cofactors (23–25).

This leaves as useful bioelements the cations of the first transition group from Mn(II) to Zn(II), which, nevertheless, also have toxic features. These metal cations fill their 3d orbitals from 3d^5^ to 3d^10^. This filling confers different chemical features to these cations, affecting both their usefulness and toxicity (Table 1). Their bond energy to first shell ligand atoms increases from Mn(II) to Cu(II), while the ionic character of the bond and the number of ligands that can be accommodated decrease. This is represented in the Irving-Williams series Mn(II) < Fe(II) < Co(II) < Ni(II) < Cu(II) > Zn(II), which describes the stability of their metal complexes (26). As noted in that study, Zn(II) falls outside the series from Cu(II) to Mn(II) and is almost on a level with Ni(II) (Table 1). The underlying chemical principles of transition metal homeostasis are more deeply explained elsewhere (1, 2) and in other recent reviews with different foci (27–29).

During the evolution of life, only resources with a benefit higher than their associated cost have been continuously used. The usefulness of metal complex-mediated biochemical reactions to the bacterial cell must minimize any potentially damaging effect mediated by transition metal cations. This is accomplished most effectively by restricting their cytoplasmic availability to a level that is just sufficient to allow correct metalation of proteins or other compounds.

## THE REQUIREMENT FOR METAL UPTAKE SYSTEMS WITH BROAD SUBSTRATE SPECIFICITY

Because the presence of a charged ion within the hydrophobic core of a biological membrane is energetically highly unfavorable, uptake systems are needed to mediate transport of transition metal cations across the inner membrane into the bacterial cytoplasm. Alternatively, metal cation may be imported as part of a complex, for instance with phosphate, histidine complexes, siderophores, or other metallophores (30–37). With respect to the uptake of metal cations, enzymes and transport systems alike have a problem in common. After binding of the respective substrate into an enzyme-substrate complex (ES), the reaction path of the respective enzymatic or transport reaction has to cross the energetic barrier of the transition complex to form the enzyme-product complex or perform the transport reaction, respectively. An increase in substrate specificity, affinity, or degree of discrimination would mean a lower energetic state of the ES, with a consequent increase in the energetic ‘distance’ to the transition complex and a lower reaction rate (38). This means that enzymes or transport systems cannot have at the same time a high substrate affinity or degree of discrimination and a high reaction rate. Because the ionic radii of the useful transition metal cations are all around 0.75 Å (Table 1) (4), a high degree of discrimination by formation of metal complexes would be needed for high-specificity uptake of a metal cation, which would strongly decrease the import rate. On the other hand, a bacterial cell needs more than 10^7^ Mg(II) ions plus up to 10^6^ cations of the other transition metals per duplication (39), so that their import rate must nevertheless be sufficiently high.

The solution to this problem seems to be a rapid and rather unspecific import of metal cations in combination with removal of surplus ions plus highly specific import at very low environmental concentrations. This is performed by a triumvirate of different categories of transport systems. A general import system (GIS) with broad substrate specificity, a process allowing high transport rates, transports Mg(II) along with divalent metal cations with similar ionic diameters, such as Co(II) and Zn(II). Among these proteins are secondary proton motive force-driven CorA-type importers of the MIT protein family (TC#1.A.35, transporter classification system [40–43]) or primary, ATP-driven MgtA-type P-type ATPases (TC#3.A.3 [44]). Removal is done by metal efflux systems that get rid of unwanted or excess ions. These exporters, however, also suffer from the transport affinity versus rate problem and cannot afford too high a substrate specificity because their overall reaction rate has to keep up with that of the general import systems. The solution is delegation of discrimination to the transcriptional regulators that control expression of the genes and operons of the respective systems. The metal-resistant beta-proteobacterium *Cupriavidus metallidurans*, for instance, possesses three P_IB2_-type (45) efflux ATPases—ZntA, CadA, and PbrA for Zn(II), Cd(II), and Pb(II), respectively—which nevertheless transport Zn(II) and Cd(II) with similar kinetic parameters (46). Each gene is regulated by its own MerR-type regulator (47–51). In this way, the regulators have sufficient time to discriminate their substrate, thereby assigning the associated efflux pump to its metal-specific export function. Consequently, correct metalation of regulatory proteins is at the core of multiple transition metal homeostasis.

The third member of the triumvirate is the ABC-type importer family (TC#3.A.1), such as ZnuABC for zinc uptake in *Escherichia coli* and many other bacteria (52), which supply metal ions to the cell especially under starvation conditions. These import systems delegate the substrate discrimination to periplasmic binding proteins (53), which, moreover, also increase the number of substrate binding sites. After binding, the substrate is delivered to the importer protein complex and transported into the cytoplasm, which is driven by ATP hydrolysis. This results in the cost of one ATP per transported ion. In comparison, the cost for a CorA-mediated import of two positive charges by the charge gradient of the proton motive force would be equal to 0.6 ATP (54) in a bacterium possessing a F_1_F_0_-ATPase with 10 c subunits. Consequently, ZnuABC is only upregulated under conditions of zinc starvation, with Zur being the responsible regulator of gene expression in many bacteria (52, 55, 56).

**Fig 1:**
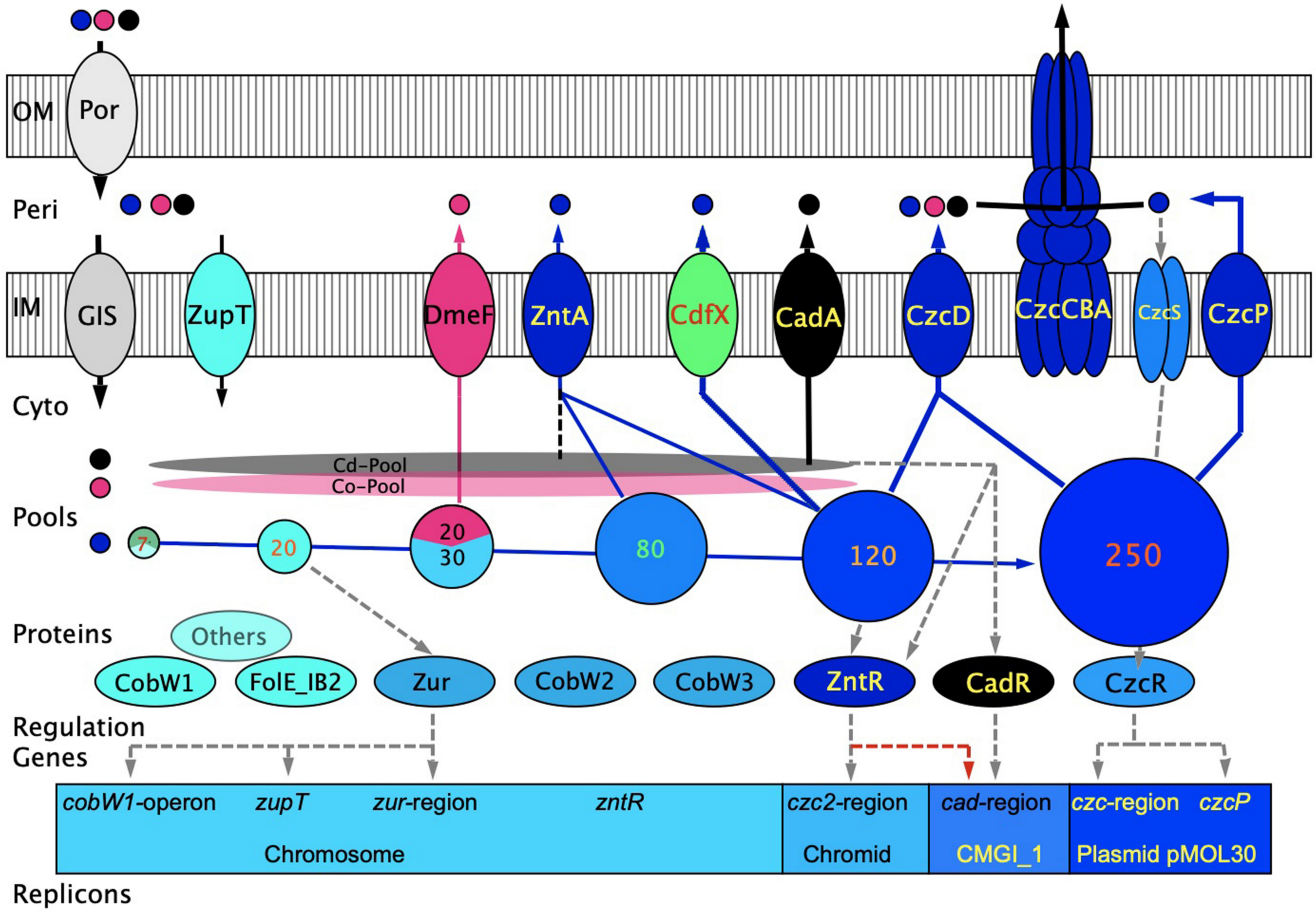
Summary of the zinc homeostasis in *C. metallidurans* CH34 under conditions of changing zinc availability. This is a revised version of a previously published figure (60). On the top and left hand, import of transition metal cations Zn(II) (blue), Co(II) (red), and Cd(II) (black) by outer-membrane (OM) porins (Por), through the periplasm (Peri) and subsequently across the inner membrane (IM) into the cytoplasm by >8 general import systems (GIS) and ZupT is depicted, leading to cytoplasmic (Cyto) zinc pools with increasing sizes from the left to the right [circles, zinc in 1,000 Zn(II) per cell] as well as Cd and Co pools (above the circles). In the cytoplasm, the ions interact with metal-binding components such as glutathione, polyphosphate, or zinc-binding sites in proteins, which form the zinc repository. Efflux systems on the top middle export these metals again (arrows). Some of these are encoded by the chromid or the genomic island CMGI_1 on the bacterial chromosome. DmeF adjusts the Co pool, while ZntA, CdfX, and CadA adjust those of Zn and Cd. At high zinc concentrations, the products of the plasmid pMOL30-encoded *czc* determinant are necessary for full metal resistance. These products provide additional efflux power for export across the IM and subsequently by the CzcCBA transenvelope complex to the outside. Products of genes on plasmid pMOL28 are not shown. At the bottom, the players in the cytoplasm are shown with dashed arrows indicating interaction and, additionally, the most important genes and the associated replicons. More details concerning the figure are in the text.

The expression of efflux systems and high-affinity import systems is inversely regulated to minimize a futile cycle of energy-dependent uptake and efflux reactions (Fig. 1). The beta-proteobacterium *C. metallidurans* requires 200 nM Zn(II) to provide sufficient zinc to the cytoplasm (57). At higher concentrations, ZntR upregulates expression of *zntA* for the efflux pump, while at lower concentrations, Zur upregulates expression of *zupT* for the main zinc uptake system (47). Nevertheless, ZupT and ZntA are both present regardless of whether there are high or low zinc concentrations in the growth medium (58, 59). This led to the hypothesis of a flow equilibrium formed by the continuous action of uptake and efflux reaction, which is central to the maintenance of the cytoplasmic zinc concentration in this bacterium and the overall composition of the transition metal complement.

## THE FLOW EQUILIBRIUM AND ITS COMPONENTS

A flow equilibrium is reached when an uptake and an efflux system transport a substrate with the same velocity (with no net release or consumption of intracellular metal stores). This leads to the formula


$$c_{i}=K_{m-ef}/(V_{max-ef}/V_{max-up}\cdot (K_{m-up}/c_{o}+1)-1)$$

with *c*_*i*_ and *c*_*o*_ being the cytoplasmic and outside concentrations of the substrate, respectively, and *K*_m_ and *V*_max_ being the kinetic parameters of the uptake and efflux systems (61). In this simple case, *c*_*i*_ could be kept constant by adaptation of the *V*_max-ef_/*V*_max-up_ ratio to the *c*_*o*_ values. Since *V*_max_ is the product of the sum of transport systems and their turnover numbers, this could be done by upregulation or downregulation of the synthesis of transport systems. This simple model indicates the best adaptation to changing *c*_*o*_ values when *c*_*o*_ is in a similar range as *K*_m-up_, for instance, between 0.1- and 10.0-folds of the *K*_m-up_. To increase this range, *C. metallidurans* contains many uptake and efflux systems with overlapping kinetic parameters (Fig. 1). Additionally, there might be flux control to quench oscillations of the flow equilibrium. Many metal transport systems contain metal-binding sites in the cytoplasm. Their occupation may downregulate the uptake rate of importers and upregulate that of exporters and may also serve here as “sponges” that concentrate the substrate metal at the exporter site (1, 2, 62–69).

It should be noted that *c*_*i*_ is not a chemical concentration as in water and should really be expressed as a chemical activity, potential or free energy. Following the Debye-Hückel law, the transition metal cations should be distributed to the anionic metal-binding sites within the cytoplasmic components by a Boltzmann distribution. This results in an apparent concentration at the metal binding site, derived from the free energy of binding a metal from an available, exchangeable, or “labile” metal pool inside the cell (29, 70–73).

Not all metals of the quota (the total number of atoms per cell, which includes every metal atom inside the cytoplasm, periplasm, cell wall, as well as cations tightly bound to proteins, or indeed the labile ones [28, 74]) are available for this exchange reaction. As shown with the periplasmic Mn(II)-binding protein MncA from a cyanobacterium, metals may be kinetically trapped during folding (70, 75–77) so that they do not exchange during a time period of metabolic significance (28). Cations present in the cytoplasm and kinetically available or those with a low probability of an exchange according to the Boltzmann distribution are available for a metalation event. The *c*_*i*_ is consequently defined by the availability of a cation for the particular efflux system that has its apparent *in vivo K*_m-ef_ in the range of this *c*_*i*_ value, because efflux systems with a lower *K*_m-ef_ should be already exporting with their *V*_max-ef_, and those with a higher *K*_m-ef_ should not contribute with significant export rates.

## THE FLOW EQUILIBRIUM AS STUDIED IN *C. METALLIDURANS*

The type strain *C. metallidurans* CH34 was isolated from a zinc decantation tank in Belgium (78). Its 6 Mb genome is organized as a chromosome, a chromid, and two large plasmids (79, 80). All four replicons carry genes involved in metal homeostasis and resistance, mostly on genomic islands or the two large plasmids, pMOL28 and pMOL30 (81–85). Central products of the plasmid-encoded *czc* and *cnr* determinants are transenvelope efflux complexes composed of (i) a trimeric resistance, nodulation, cell division (RND) (86–90) protein in the cytoplasmic membrane and extending into the periplasm; (ii) an outer membrane factor (OMF) (91–93) that forms one beta-barrel pore per trimer in the outer membrane and also extends into the periplasm; and (iii) a hexameric membrane fusion or adapter protein that connects the RND and OMF proteins (86, 94–100). Although the RND protein may export metal cations *in vitro* from artificial systems that are proxies of the cytoplasm (90, 93), there is no evidence for such a process *in vivo*. These transenvelope efflux systems export their substrates from the periplasm to the outside (46, 101–103). Export from the periplasm to the outside has been shown directly for another group of RND-driven efflux systems for organic substances (104).

Pulse-chase experiments using radioactive ^65^Zn and isotope-enriched stable ^67^Zn provided evidence that uptake and efflux reactions were indeed occurring in parallel in *C. metallidurans* (105), forming a flow equilibrium of Zn(II) in these cells. To avoid interference with the plasmid-encoded RND-driven transenvelope efflux systems, these experiments were done with the plasmid-free strain AE104 and several mutants of this parent strain. An efflux during the chase was also observed when other metal cations were used instead of zinc, in the order Zn(II) > Co(II) > Ni(II) > Mn(II) > Mg(II) or 100 µM EDTA instead of a chasing metal, which hinders ^65^Zn uptake by sequestration of the cation (105). Decreasing the zinc concentration to starvation conditions during cultivation of the cells used for the pulse-chase experiments did not change the outcome. But an additional decrease of the Mg(II) concentration in the growth medium from 1 mM to 100 µM increased the zinc uptake rate during the pulse and even led to a slow efflux in the un-chased control. Moreover, the Mg(II) used for the chase resulted in a stronger zinc efflux in magnesium-starved cells than in the control cells (105). This provided evidence that indeed uptake systems with a broad substrate range were operating in *C. metallidurans*, for instance, magnesium uptake systems.

A variety of metal uptake systems with such a broad substrate specificity (GIS, Fig. 1) are involved in the uptake of Zn(II) by *C. metallidurans* (39, 106–108). These include (i) the four proteins CorA_1_, CorA_2_, CorA_3_, and ZntB (42, 43, 65, 109) of the MIT protein family (TC 1.A.35) that are mainly Mg(II) importers; (ii) the PitA phosphate-metal-proton symporter (PiT family, TC 2.A.20) (36, 37); (iii) the HoxN Ni(II) importer (NiCoT family, TC 2.A.52) (110, 111); and (iv) two P-type Mg/Ca importers, MgtA and MgtB (TC 3.A.3) (42, 112). All of these uptake systems contribute to the import of zinc in this bacterium (39, 106–108, 113).

*C. metallidurans* does not have a ZnuABC importer (39). Instead of *znuABC*, Zur in *C. metallidurans* regulates expression of the *zupT* gene for an importer of the ZIP protein family (TC 2.A.5) (39, 114–119). Deletion of *zupT* influences the zinc pool in *C. metallidurans*. Synthesis of the zinc-dependent RpoC subunit of the RNA polymerase is disturbed, leading to accumulation of RpoC proteins in inclusion bodies (113). Moreover, production of the CzcCBA transenvelope efflux system is prevented, for instance, by curing of the *czc*-carrying plasmid pMOL30 as published (113). ZupT should be an important zinc importer in *C. metallidurans* (Fig. 1). Indeed, compared to the parent AE104, zinc uptake during the pulse was on a lower level in the ∆*zupT* mutant, especially in cells grown under zinc- and magnesium-starvation conditions (105). Mutant strain ∆7 with additional deletions of *corA_1, 2, 3_*, *zntB*, *pitA*, and *hoxN* imported zinc with even lower rates. Surprisingly, an additional deletion of *mgtA* and *mgtB* for the two P-type ATPases (∆7 ∆*mgtA ∆mgtB*) resulted in the ∆9 mutant strain, which again imported Zn(II) with a higher rate than the ∆7 or ∆*zupT* strains but only when the cells were cultivated with ambient metal supply. This indicated the presence of an unknown zinc importer “X” that functions more efficiently in cells grown under ambient metal supply than in metal-starved cells (105).

Cytoplasmic metal-binding components should affect the availability of metal cations for the regulators of gene expression of the transport systems and possibly the flux control mechanisms. Removal of glutathione (∆*gshA*) or of polyphosphate (∆*ppk*) indeed decreased metal uptake, especially in metal-starved cells. Polyphosphate, a “hard” Lewis base, had a stronger effect in cells starved for the hard Lewis acid Mg(II) than the soft thiol compound glutathione. Neither compound affected the efflux reaction (105). This indicated that there is an important contribution of cytoplasmic metal buffers to the cellular metal homeostasis, e.g., glutathione and polyphosphate, in addition to the general zinc repository or metal-binding proteins (2, 120).

The presence of efflux systems should also influence the flow equilibrium because they counteract the activity of the uptake systems. In the plasmid-free *C. metallidurans* strain AE104, at least eight efflux systems can be involved in transport of the ion across the inner membrane, the three CDF proteins (TC 2.A.4) (121), DmeF mainly for Co(II), FieF for Fe(II), and CdfX for Zn(II) plus three Cu(I)-exporting P_IB1_-type ATPases and two P_IB2_-type ATPases for Zn(II) and Cd(II) (45, 46, 60, 103, 122–124). Deletion of the genes encoding the two zinc-cadmium-exporting P_IB2_-type ATPases, *zntA* and *cadA*, in the ∆e2 mutant decreased the import rate of zinc and the subsequent efflux rate during the chase. Although DmeF and FieF do not contribute much to zinc and cadmium resistance (46), the zinc uptake and efflux rates were more strongly decreased in the ∆e4 (∆e2 ∆*dmef ∆fieF*) than in the ∆e2 mutant (105).

Residual efflux activity in the ∆e4 mutant strain led to the identification of CdfX as another efflux system, which is involved in zinc export and homeostasis (60). Expression of the *cdfX* gene is under zinc control via ZntR (Fig. 1), which also controls expression of *zntA*. ZntA is the main efflux system of cytoplasmic Zn(II) and Cd(II). When the Cd(II) concentration becomes too high for an efficient cadmium removal by ZntA, the *cadA* gene for cadmium-exporting CadA is expressed under cadmium control by CadR. When, on the other hand, the zinc content in a cytoplasmic Cd/Zn mixture becomes too high for an efficient zinc removal by ZntA, CdfX is produced to solve this problem. In this way, zinc and other ions are efficiently removed from the cytoplasm (Fig. 1), and the cytoplasmic steady state is kept in a flow equilibrium (105, 125).

The pulse-chase experiments with radioactive ^65^Zn(II) and stable enriched ^67^Zn(II) (105) indicated that indeed a flow equilibrium of Zn(II) ions seems to exist in *C. metallidurans*, which was formed by the continuous parallel action of general import systems, which are mainly for Mg(II) ions, plus ZupT, zinc efflux systems, and a metal-buffering effect by glutathione and polyphosphate. Since both transport processes are energy dependent, a futile cycle seemed to be formed, but the respective energy is used to adjust the cytoplasmic metal cation complement to minimize mis-metalation along the Irving-Williams series (125). The composition and availability of the cytoplasmic metal cations are thus in a steady state that is formed by a flow equilibrium of these import and export processes.

## TRANSITION METALS AS ESSENTIAL-BUT-TOXIC AND COMPETING CATIONS

Once inside the bacterial cell, Mg(II) and transition metal cations pass between Lewis bases following the Debye-Hückel law until a metal-specific protein needs to be metalated. Mn(II) with 3d^5^ can be stable in many oxidation states, which allows its most important function as the central component of the water-splitting complex in photosystem II of cyanobacteria (126) or of Mn-dependent superoxide dismutase (127). Mn(II) is not used by *C. metallidurans*, which has no uptake system of the NRAMP protein family and no Mn-dependent superoxide dismutase, probably to prevent Cd(II) uptake because NRAMP systems are notorious Cd(II) importers (39, 128–132). In *E. coli*, Mn(II) is used to substitute Mn(II) for Fe(II) under oxidative stress (133, 134) so that the Mn content of *E. coli* under non-stress conditions is also very low.

Fe is the most important minor bioelement in most bacteria. With 3d^6^ as Fe(II) and 3d^5^ in Fe(III), iron is central to redox reactions that transfer a single electron. *C. metallidurans* contains half a million Fe atoms per cell (39), used for cytochromes, mononuclear iron centers, and iron-sulfur proteins such as aconitase. Under oxic conditions, the prominent Fe(III) forms insoluble hydroxide complexes at a neutral pH value so that bacteria produce siderophores to sequester Fe(III) and provide it to the cell (135). When growing in the lab, *C. metallidurans* uses iron citrate provided in the mineral salt medium, while it additionally produces the siderophore, alcaligin E (31, 136, 137). Inside cells, iron is rapidly reduced to Fe(II), which can result in the Fenton reaction producing reactive oxygen species that damage biological macromolecules, even when the iron is in iron-sulfur centers (12, 138, 139). Bacterioferritins and Dps oxidize Fe(II) to Fe(III) stored inside these proteins (140, 141) so that the quota of half a million Fe atoms per cell probably does not reflect the cytoplasmic availability of iron. Moreover, cytoplasmic Fe(II) can also be removed by efflux systems (122, 123, 142, 143).

*C. metallidurans* contains only 4,000–5,000 Co (3d^7^) cations per cell when cultivated under Zn-replete conditions (39, 57), and Co(II) has also a low availability in most environments, such as sea water (4). At higher concentrations, it may interfere with the iron metabolism, causing damage during *de novo* synthesis of iron-sulfur centers or mis-metalation of protoporphyrin IX (8–11). Cobalt is mainly used bound to cobalamin compounds in mutases, which rearrange C-C and C-H bonds (144). To solve the problem of the generally low availability of cobalt in the environment, combined with a high risk of causing an imbalance in iron homeostasis should the cytoplasmic cobalt content increase excessively, cobalamin is used as a kind of “cobalto-phore” and exchanged between cobalamin-producing and cobalamin-utilizing bacteria. While Fe(II), Fe(III), and Co(III) can form stable octahedral complexes, Ni(II) with 3d^8^ is not able to do so and forms square planar complexes in many instances (145–148). Ni(II) may interfere with Fe(II) and Zn(II) homeostasis (14, 15), and while its cytoplasmic quota is kept low, it is used in aerobic bacteria only for a few enzymes such as urease, hydrogenase, and a nickel-dependent superoxide dismutase (149–152).

Cu appears as Cu(II) (3d^9^) in oxic environments but is immediately reduced to Cu(I) (3d^10^) in the cytoplasm or upon contact with respiratory chain components already in the periplasm (153, 154). It is used in a variety of enzymes that interact directly with molecular oxygen, such as in cytochrome *c* oxidase. As the only soft transition metal cation of the first transition period, its toxicity in the cytoplasm is based upon binding to sulfur atoms in iron-sulfur clusters (17), also during their assembly (19, 155), and to thiols of proteins causing impaired folding (156). Under oxic conditions in the periplasm, but not the cytoplasm (134), Cu(I) causes oxidative damage and inhibits assembly of *c*-type cytochromes (157) so that availability of Cu(I) has to be kept extremely low in both compartments (158, 159). This is mediated by many interacting reactions (103, 160–162): (i) binding to chaperones in the cytoplasm (163–165), (ii) efflux into the periplasm by P_IB1_-type ATPases (166–168), (iii) efflux to the outside of the cell by the CusCBA transenvelope complex (169–171), and (iv) oxidation to the less toxic Cu(II) in the periplasm (172–176).

Zn(II) with a completely filled 3d^10^ orbital cannot be reduced or oxidized under physiological conditions and is only able to accommodate four ligands in tetrahedral complexes. Bound Zn(II) can be used as structural zinc to stabilize a protein's conformation or, with one ligand position open, for Lewis acid-mediated catalytic reactions (177). *C. metallidurans* keeps its zinc quota at about 80,000 Zn per cell, which is second in ranking to the number of Fe atoms (39, 57). In contrast, *C. metallidurans* possesses about 120,000 copies of potential zinc-binding proteins (120), so that even under supply of sufficient zinc, zinc-binding sites remain available for immediate occupation.

An important zinc-dependent protein is a FolE_IA GTP-cyclohydrolase that initiates folate synthesis. Since formyl-tetrahydrofolate is also essential for GTP biosynthesis, a *cyclus diabolus* exists between GTP and folate. *Bacillus subtilis* contains two FolE-type enzymes, a zinc-dependent FolE_IA and a metal-promiscuous FolE_IB (178), while *C. metallidurans* has three FolE-type enzymes. FolE_IA is strictly zinc dependent and is needed in the presence of Cd(II), metal chelators, and hydrogen peroxide. FolE_IB1 and FolE_IB2 depend on Fe(II), Mn(II), and Co(II), with FolE_IB1 used under zinc-starvation conditions and FolE_IB2 used under low zinc and cobalt but high magnesium availability (179). As *C. metallidurans* does not possess a manganese uptake system and contains only a few hundred Mn atoms per cell (39, 179), the main cofactors for the two FolE_IBs are Fe(II) or Co(II). In contrast to FolE_IB1 and FolE_IB2, the metal cofactor could not be released from FolE_IA to form a zinc-free apoenzyme by treatment with metal-complexing compounds. This indicates that the Zn(II) is kinetically trapped in FolE_IA, excluding a “hop-on-hop-off” metalation as in the case of the FolE_IBs (179).

Another important zinc-dependent protein is the beta-prime subunit RpoC of the bacterial RNA polymerase with Zn(II) bound firmly by four cysteine residues (180–182). Correct metalation of RpoC is determined by the omega subunit RpoZ, which allows introduction of Zn(II) into the apo protein only when RpoC is correctly folded (183). This indicates that in RpoC, FolE_IA, and possibly in other proteins, Zn(II) is kinetically trapped and is likely to be inserted immediately after translation. When a portion of the 120,000 proteins with zinc-binding sites in *C. metallidurans* trap the metal kinetically, another portion of these proteins presumably has a zinc repository function. Examples could include zinc-binding sites in the ribosome and other subunits of the RNA polymerase (180, 181, 184–186) or the COG0523 protein CobW2, which serves as a zinc-storage protein in *C. metallidurans* (57, 187).

## MIS-METALATION AND THE CONTROL OF THE TRANSITION METAL CATION POOLS

The flow equilibrium adjusts the cell-bound quota of metals in *C. metallidurans* to 14,000,000 Mg > 537,000 Fe > 91,000 Zn > 61,000 Cu > 11,000 Ni > 4,000 Co > 993 Mn (39). Only a portion of these ions is available for metalation or mis-metalation reactions and is a substrate for the respective efflux system. This portion also governs metal homeostasis, either by interacting with regulators such as ZntR that increase the number of efflux systems and subsequently their overall *V*_max-ef_ activities, or by regulators that upregulate the number of import systems, e.g., Zur. In this way, toxicity resulting from an uncalibrated metal cation pool is prevented.

Consequently, regulatory processes that adjust the number and activity of transport proteins are responsible for the maintenance of the cytoplasmic transition metal cation pools. These include the metal-binding sites of the transport proteins that may be involved in flux control (63, 67, 188, 189) or riboswitches (190), but most important are the regulators of gene expression (55, 74, 159, 191–198). They have to perform the dual task of metal discrimination and determination of their availability (28). Regulators have a primary metal-binding site required for discrimination; sometimes they have additional allosteric sites or sites important for their structural integrity (199). The first shell atoms of the primary site are central for the correct discrimination of a transition metal cation. For example, Mn(II) and Fe(II) sensors use O and N but not S; Mn(II) predominantly uses O, which agrees with Mn(II) being a hard Lewis acid; and Fe(II) uses O and N equally. For the divalent cations Co(II), Ni(II), and Zn(II), N is the most important first shell ligand, but S atoms are also used. Finally, Cu(I) mainly interacts with S (199).

The number of possible ligands and the geometry of the complexes, octahedral, distorted octahedral, or tetrahedral, are additionally used to discriminate between these metals. This selective feature directly depends on the number of their 3d electrons (146, 147, 199). Shielding effects occur with an increasing number of ligands, especially for Zn(II) and Mn(II), which cause a negative electron affinity (1, 2, 199). Finally, allosteric effects and completeness of the assembly of the binding site may be used to increase selectivity of metal binding (199). These mechanisms organize metal-specific upregulation of the uptake systems, particularly when starvation conditions are imposed, or alternatively, they induce synthesis of efflux systems when the metal-dependent toxic burden increases (47, 55, 197).

However, regulators can also be mis-metalated when the composition of the overall cytoplasmic metal cation complement is disturbed, for instance, by binding of Co(II) to the iron uptake regulator Fur (70, 200). This mis-metalation can be simulated in *E. coli* using the “metalation calculator” (201). The data predicted here have been experimentally verified using the Mn(II)-binding protein MncA, which kinetically traps the metal bound during folding (70). These data demonstrate very clearly that correct homeostatic control of the cytoplasmic transition metal complement is a prerequisite to prevent mis-metalation of proteins (29) or regulators (73) that close the feedback regulatory loop between flow equilibrium, metal-pool adjustment, and metalation (125).

Moreover, these findings using metalation calculators (70) imply that inter-metal competition from exchangeable metal pools to proteins approximates thermodynamic equilibrium, at least when the respective flow equilibria are at steady state. This is because predictions of the metalation calculators are based on a thermodynamic equilibrium model and assume rapid associative metal exchange at this step. Other aspects of metal homeostasis that precede or follow this exchange step, including metal-transport, metal-trafficking, and protein folding, can be non-equilibrium and dominated by kinetics. Importantly, the flow equilibrium model and metalation calculators (https://metalation-calculator.awh.durham.ac.uk/) describe different steps and are compatible.

## *C. METALLIDURANS:* MODEL SYSTEM OR EXCEPTION?

A model (Fig. 1) summarizing the data primarily for zinc homeostasis in *C. metallidurans* has been published (60). The flow equilibrium (105) is formed by GIS action (Fig. 1), ZupT plus ZntA with *zupT* being under Zur control and *zntA* under the control of ZntR (47). Related proteins are present in many bacteria, but most of these bacteria have an additional ZnuABC import system (52, 117, 202) so that a flow equilibrium probably also exists for these microorganisms.

Three proteins belonging to the COG0523 family of proteins—CobW1, CobW2, and CobW3—are also involved in zinc homeostasis in *C. metallidurans* (105, 118, 187). Related proteins deliver Ni(II) to hydrogenases and the urease. Initially found in the vicinity of cobalamin biosynthesis operons, the genes for these COG0523 proteins are often under Zur control, which is also the case in *C. metallidurans*. Zur control indicates a function under zinc starvation conditions (203). These proteins are present in many organisms from bacteria to vertebrates and plants (178, 201, 204–207). They may deliver Zn(II) to zinc-dependent proteins with the energy of GTP hydrolysis used to dissociate the protein-protein interaction.

While CobW1 in *C. metallidurans* resembles ZagA (178) with one zinc-binding site close to the site controlling GTP hydrolysis, CobW2 contains a sequence of numerous and adjacent metal-binding sites in the middle of the protein. The protein occurs in two different conformations and increases the number of cell-bound zinc ions. CobW2 may be a zinc-storage compound that releases Zn(II) upon a conformational change. CobW3 also contains a sequence of adjacent metal-binding sites but located at the carboxy-terminus. CobW3 has lost its GTPase function and may instead control the activity of metal transport systems (118, 187). Both proteins affect the flow equilibrium of zinc in pulse-chase experiments (105).

The substitution of some of the cell-bound zinc by cobalt under zinc-starvation conditions (57), for instance, to activate FolE_IB-type enzymes (179) (Fig. 1) has been previously shown for *Salmonella* (208). The process requires in *C. metallidurans* an interaction of ZupT with CobW2 and CobW3 and export of Co(II) by DmeF when the cytoplasmic cobalt concentration becomes too high (57). This means that these CobW proteins are involved not only in zinc but also in cobalt homeostasis (201), with CobW2 and CobW3 being at the crossroads of both processes in *C. metallidurans* (57) (Fig. 1). Since CobW-like COG0523 proteins occur in many organisms (178, 201, 204–207), such a Zn-Co cross-link may also be a widespread phenomenon.

At micromolar concentrations of Zn(II) and Cd(II) (Fig. 1), ZntA is able to export both cations from the cytoplasm (46, 209) and is supported by CadA or CdfX when the availability of cytoplasmic Cd(II) or Zn(II) increases, respectively (60). The *czc* determinant on plasmid pMOL30 is responsible for even higher zinc concentrations and exports Zn(II) across the inner membrane to the periplasm by the CDF protein CzcD and the P_IB4_-type ATPase CzcP for subsequent export across the outer membrane by CzcCBA (Fig. 1) (46, 96, 106, 210).

The *czc* determinant in *C. metallidurans* is unique in its complexity with the components of the transenvelope efflux systems, periplasmic proteins, CzcD, CzcP, and a two-component regulatory system; however, related determinants are also widespread in other bacteria (88). The identified mechanisms of transition metal homeostasis should, therefore, also be present in other bacteria but perhaps in a simpler form. Concerning zinc homeostasis (Fig. 1), the left-hand half of the scheme (sufficient zinc and zinc starvation) can serve as a general model for many bacteria. Moving to the right hand into the domain of high-level zinc resistance, the situation becomes more and more specific for *C. metallidurans*.
